# *N*′-[(*E*)-1-(2-Fluoro­phen­yl)ethyl­idene]pyridine-4-carbohydrazide

**DOI:** 10.1107/S1600536814007545

**Published:** 2014-04-09

**Authors:** P. B. Sreeja, M. Sithambaresan, N. Aiswarya, M. R. Prathapachandra Kurup

**Affiliations:** a Department of Chemistry, Christ University, Hosur Road, Bangalore 560 029, Karnataka, India; b Department of Chemistry, Faculty of Science, Eastern University, Sri Lanka, Chenkalady, Sri Lanka; c Department of Applied Chemistry, Cochin University of Science and Technology, Kochi 682 022, India

## Abstract

The title compound, C_14_H_12_FN_3_O, adopts an *E* conformation with respect to the azomethine bond. The pyridyl and fluoro­benzene rings make dihedral angles of 38.58 (6) and 41.61 (5)° respectively with the central C(=O)N_2_CC unit, resulting in a non-planar mol­ecule. The inter­molecular inter­actions comprise two classical N—H⋯O and N—H⋯N hydrogen bonds and four non-classical C—H⋯O and C—H⋯F hydrogen bonds. These inter­actions are augmented by a weak π–π inter­action between the benzene and pyridyl rings of neighbouring mol­ecules, with a centroid–centroid distance of 3.9226 (10) Å. This leads to a three-dimensional supra­molecular assembly in the crystal system. The F atom is disordered over two sites in a 0.559 (3): 0.441 (3) ratio, through a 180° rotation of the fluoro­benzene ring.

## Related literature   

For biological properties of hydrazones, see: Kahwa *et al.* (1986 ▶); Santos *et al.* (2001 ▶); Rollas & Kucukguzel (2007 ▶). For the synthesis of related compounds, see: Mangalam & Kurup (2011 ▶). For related structures, see: Sreeja *et al.* (2013 ▶, 2014 ▶).
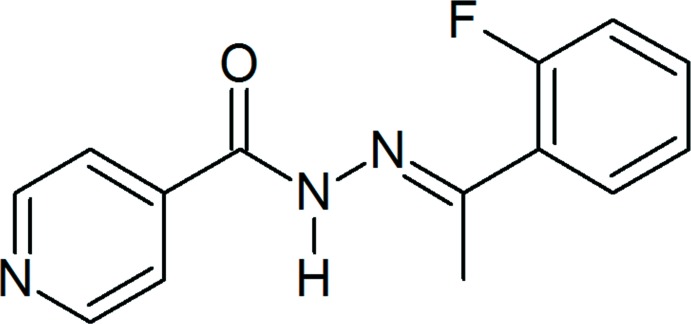


## Experimental   

### 

#### Crystal data  

C_14_H_12_FN_3_O
*M* *_r_* = 257.27
Monoclinic, 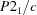

*a* = 8.2649 (6) Å
*b* = 19.2127 (14) Å
*c* = 8.0554 (5) Å
β = 99.244 (3)°
*V* = 1262.51 (15) Å^3^
*Z* = 4
Mo *K*α radiation
μ = 0.10 mm^−1^
*T* = 296 K
0.35 × 0.30 × 0.25 mm

#### Data collection  

Bruker Kappa APEXII CCD diffractometer
Absorption correction: multi-scan (*SADABS*; Bruker, 2004 ▶) *T*_min_ = 0.965, *T*_max_ = 0.976
9610 measured reflections
3137 independent reflections
2262 reflections with *I* > 2σ(*I*)
*R*_int_ = 0.030

#### Refinement  

*R*[*F* ^2^ > 2σ(*F* ^2^)] = 0.048
*wR*(*F* ^2^) = 0.152
*S* = 1.04
3113 reflections
179 parameters
1 restraint
H atoms treated by a mixture of independent and constrained refinement
Δρ_max_ = 0.24 e Å^−3^
Δρ_min_ = −0.21 e Å^−3^

### 

Data collection: *APEX2* (Bruker, 2004 ▶); cell refinement: *APEX2* and *SAINT* (Bruker, 2004 ▶); data reduction: *SAINT* and *XPREP* (Bruker, 2004 ▶); program(s) used to solve structure: *SHELXS97* (Sheldrick, 2008 ▶); program(s) used to refine structure: *SHELXL97* (Sheldrick, 2008 ▶); molecular graphics: *ORTEP-3 for Windows* (Farrugia, 2012 ▶) and *DIAMOND* (Brandenburg, 2010 ▶); software used to prepare material for publication: *SHELXL97* and *publCIF* (Westrip, 2010 ▶).

## Supplementary Material

Crystal structure: contains datablock(s) Global, I. DOI: 10.1107/S1600536814007545/fj2669sup1.cif

Structure factors: contains datablock(s) I. DOI: 10.1107/S1600536814007545/fj2669Isup2.hkl

Supporting information file. DOI: 10.1107/S1600536814007545/fj2669Isup3.cml

CCDC reference: 995414

Additional supporting information:  crystallographic information; 3D view; checkCIF report

## Figures and Tables

**Table 1:** Hydrogen-bond geometry (Å, °)

*D*—H⋯*A*	*D*—H	H⋯*A*	*D*⋯*A*	*D*—H⋯*A*
N2—H2′⋯N1^i^	0.87 (1)	2.45 (1)	3.1420 (15)	137 (1)
N2—H2′⋯O1^i^	0.87 (1)	2.38 (1)	3.1777 (15)	154 (2)
C8—H8*C*⋯F1^i^	0.96	2.46	3.1603 (19)	129
C8—H8*C*⋯O1^i^	0.96	2.58	3.0680 (13)	112
C13—H13⋯F1^ii^	0.93	2.34	3.238 (2)	161
C14—H14⋯O1^i^	0.93	2.50	3.1849 (19)	131

Symmetry codes: (i) 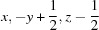; (ii) 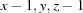.

**Fig. 1.**
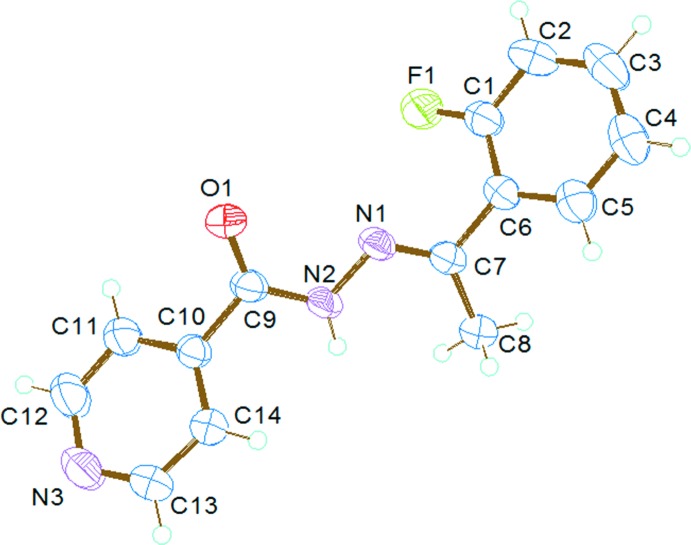
ORTEP diagram of N'-[(1E)-1-(2-fluorophenyl)ethylidene]pyridine-4-carbohydrazide with 50% probability ellipsoids. The minor components of fluorine and hydrogen atoms of the disorder are omitted.

**Fig. 2.**
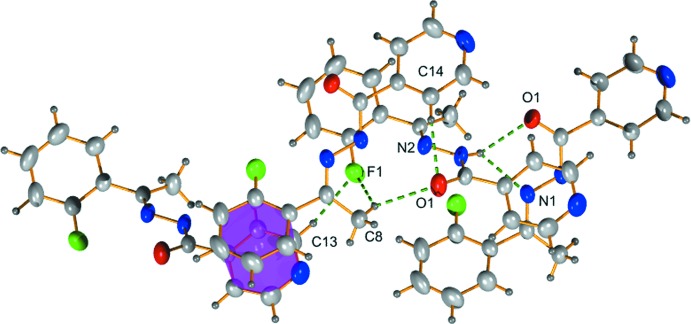
Hydrogen-bonding and π···π interactions in the title compound. The minor components of fluorine and hydrogen atoms of the disorder are omitted.

**Fig. 3.**
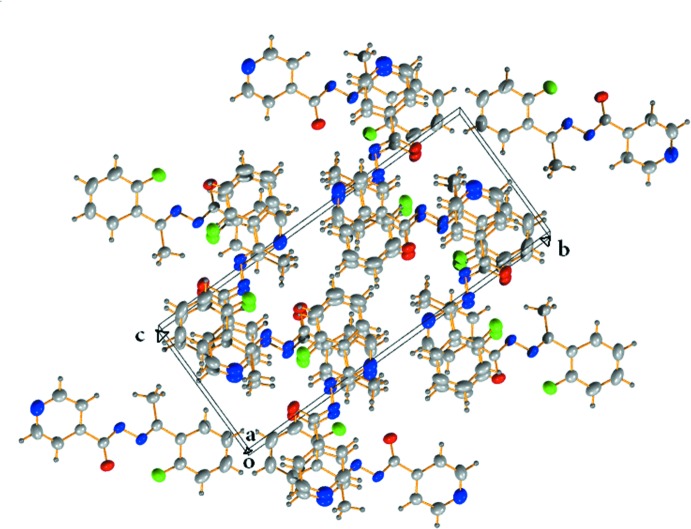
Packing diagram of the title compound along a axis.

## supplementary crystallographic information

### 1. Comment

The chemistry of Schiff bases has attracted a great deal of interest in recent years. These compounds play an important role in the development of various proteins and enzymes (Kahwa *et al.*, 1986; Santos *et al.*, 2001). A number of hydrazones derived from isoniazid were reported to be active antitubercular agents and were found to be less toxic than isoniazid (Rollas & Kucukguzel, 2007). In this paper we report the synthesis and crystal structure of the title compound.

The molecule crystallizes in monoclinic space group *P*2_1_/c. The compound adopts an *E* configuration with respect to the azomethine olefinic bond whilst the C8 and N2 atoms are in *Z* configuration with respect to the same bond with torsion angles of -177.77 (8) of and 2.13 (14)° respectively (Fig. 1). The ketonic O and the azomethine N are also *cis* to each other with a torsion angle of 1.4 (2)°. The molecule exists in the amido form with a C9=O1 bond length of 1.2208 (16) Å which is very close to the reported C=O bond length of a similar structure (Sreeja *et al.*, 2013). The pyridyl ring and the fluorophenyl ring make a dihedral angle of 38.58 (6) and 41.61 (5)° with the C(=O)N_2_CC central unit making the molecule non-planar.

There exist two classical N–H···O and N–H···N hydrogen bonding interactions with D···A distances of 3.1777 (15) and 3.1419 (15) Å respectively (Table 1). In addition to this, there are four non-classical C–H···O and C–H···F H bonding interactions present with D···A distances of 3.1603 (19), 3.0680 (13), 3.238 (2) and 3.1849 (19) Å connecting various adjacent molecules together with the main molecule (Fig. 2). The hydrogen atoms at N2 and C8 form bifurcated hydrogen bonds with O1 & N1 and F1 and O1 respectively (Fig. 2). A weak π···π interaction between the phenyl and the pyridyl ring of the neighbouring molecules also supports to form a three-dimensional supramolecular assembly together with the dominant H bonding interactions with a centroid-centroid distance of 3.9226 (10) Å (Fig. 2). Fig. 3 shows the packing of the molecules by means of hydrogen bonding and π–π interactions along *a* axis.

Through a 180° rotation of the fluorophenyl ring, the fluorine atom F1 is disordered over two sites in a ratio of 56.0 (1):44.0 (1). Similar instances of positional disorder had been previously reported (Sreeja *et al.*, 2014).

### 2. Experimental

The title compound was prepared by adapting a reported procedure (Mangalam & Kurup, 2011). Methanolic solutions of pyridine-4-carbohydrazide (0.137 g, 1 mmol) and 1-(2-fluorophenyl)ethanone (0.138 g, 1 mmol) was refluxed, in presence of a few drops of glacial acetic acid for 6 h. On cooling the reactant media, colourless crystals of hydrazones were separated out. The crystals were filtered and washed with minimum quantity of methanol and dried over P_4_O_10_*in vacuo*. Good quality block shaped crystals suitable for X-ray analysis, were obtained from methanolic solution by slow evaporation.

### 3. Refinement

The fluorine atoms F1 and F1B of this molecule were refined freely, with the sum of their occupancy factors constrained to 1.0. The H5 at C5 atom is placed in geometrically idealized position with occupancy factor equal to that F1, and its coordinates were fixed. The H1 atom was refined with restrained distance of 0.93 Å with occupancy factor equal to that of F1B. The N2—H2' distance was restrained to 0.88±0.01 Å. The H atoms on the rest of C atoms were placed in calculated positions, guided by difference maps, with C–H bond distances 0.93–0.96 Å. H atoms were assigned as *U*_iso_(H)=1.2Ueq(carrier) or 1.5Ueq (methyl C).

### Crystal data

**Table:**

C_14_H_12_FN_3_O	*F*(000) = 536
*M**_r_* = 257.27	*D*_x_ = 1.354 Mg m^−^^3^
Monoclinic, *P*2_1_/*c*	Mo *K*α radiation, λ = 0.71073 Å
Hall symbol: -P 2ybc	Cell parameters from 3938 reflections
*a* = 8.2649 (6) Å	θ = 2.5–28.2°
*b* = 19.2127 (14) Å	µ = 0.10 mm^−^^1^
*c* = 8.0554 (5) Å	*T* = 296 K
β = 99.244 (3)°	Block, colorless
*V* = 1262.51 (15) Å^3^	0.35 × 0.30 × 0.25 mm
*Z* = 4	

### Data collection

**Table:**

Bruker Kappa APEXII CCD diffractometer	3137 independent reflections
Radiation source: fine-focus sealed tube	2262 reflections with *I* > 2σ(*I*)
Graphite monochromator	*R*_int_ = 0.030
Detector resolution: 8.33 pixels mm^-1^	θ_max_ = 28.3°, θ_min_ = 2.5°
ω and φ scan	*h* = −10→10
Absorption correction: multi-scan (*SADABS*; Bruker, 2004)	*k* = −25→25
*T*_min_ = 0.965, *T*_max_ = 0.976	*l* = −6→10
9610 measured reflections	

### Refinement

**Table:**

Refinement on *F*^2^	Secondary atom site location: difference Fourier map
Least-squares matrix: full	Hydrogen site location: inferred from neighbouring sites
*R*[*F*^2^ > 2σ(*F*^2^)] = 0.048	H atoms treated by a mixture of independent and constrained refinement
*wR*(*F*^2^) = 0.152	*w* = 1/[σ^2^(*F*_o_^2^) + (0.0851*P*)^2^ + 0.152*P*] where *P* = (*F*_o_^2^ + 2*F*_c_^2^)/3
*S* = 1.04	(Δ/σ)_max_ < 0.001
3113 reflections	Δρ_max_ = 0.24 e Å^−^^3^
179 parameters	Δρ_min_ = −0.21 e Å^−^^3^
1 restraint	Extinction correction: *SHELXL*, Fc^*^=kFc[1+0.001xFc^2^λ^3^/sin(2θ)]^-1/4^
Primary atom site location: structure-invariant direct methods	Extinction coefficient: 0.091 (9)

### Special details

**Table:**

Geometry. All e.s.d.'s (except the e.s.d. in the dihedral angle between two l.s. planes) are estimated using the full covariance matrix. The cell e.s.d.'s are taken into account individually in the estimation of e.s.d.'s in distances, angles and torsion angles; correlations between e.s.d.'s in cell parameters are only used when they are defined by crystal symmetry. An approximate (isotropic) treatment of cell e.s.d.'s is used for estimating e.s.d.'s involving l.s. planes.
Refinement. Refinement of *F*^2^ against ALL reflections. The weighted *R*-factor *wR* and goodness of fit *S* are based on *F*^2^, conventional *R*-factors *R* are based on *F*, with *F* set to zero for negative *F*^2^. The threshold expression of *F*^2^ > σ(*F*^2^) is used only for calculating *R*-factors(gt) *etc*. and is not relevant to the choice of reflections for refinement. *R*-factors based on *F*^2^ are statistically about twice as large as those based on *F*, and *R*- factors based on ALL data will be even larger.

### Fractional atomic coordinates and isotropic or equivalent isotropic displacement parameters (Å^2^)

**Table:**

	*x*	*y*	*z*	*U*_iso_*/*U*_eq_	Occ. (<1)
F1	1.2954 (2)	0.23496 (9)	0.7843 (2)	0.0687 (7)	0.559 (3)
F1B	1.1039 (4)	0.01885 (12)	0.6026 (3)	0.0868 (11)	0.441 (3)
O1	0.85862 (15)	0.33623 (6)	0.55332 (12)	0.0580 (3)	
N1	1.02285 (13)	0.21790 (6)	0.53744 (12)	0.0407 (3)	
N2	0.93699 (14)	0.24882 (6)	0.39513 (13)	0.0412 (3)	
N3	0.5574 (2)	0.40011 (8)	−0.01035 (19)	0.0675 (4)	
C1	1.27207 (19)	0.16966 (8)	0.80753 (18)	0.0496 (4)	
H1	1.2797	0.2175	0.7923	0.059*	0.441 (3)
C2	1.3481 (2)	0.14096 (11)	0.9566 (2)	0.0674 (5)	
H2	1.4056	0.1691	1.0399	0.081*	
C3	1.3382 (3)	0.07038 (11)	0.9809 (2)	0.0726 (5)	
H3	1.3889	0.0505	1.0811	0.087*	
C4	1.2544 (3)	0.02970 (10)	0.8585 (3)	0.0734 (5)	
H4	1.2473	−0.0181	0.8746	0.088*	
C5	1.17979 (10)	0.05957 (4)	0.71021 (9)	0.0592 (4)	
H5	1.1236	0.0310	0.6271	0.071*	0.559 (3)
C6	1.18520 (10)	0.13048 (4)	0.68017 (9)	0.0411 (3)	
C7	1.10140 (10)	0.16188 (4)	0.52091 (9)	0.0399 (3)	
C8	1.11560 (10)	0.12603 (4)	0.35878 (9)	0.0566 (4)	
H8A	1.0211	0.0972	0.3257	0.085*	
H8B	1.2126	0.0977	0.3735	0.085*	
H8C	1.1222	0.1602	0.2732	0.085*	
C9	0.85653 (17)	0.30834 (7)	0.41681 (15)	0.0409 (3)	
C10	0.75600 (16)	0.33873 (7)	0.26301 (16)	0.0390 (3)	
C11	0.7454 (2)	0.40996 (8)	0.2466 (2)	0.0546 (4)	
H11	0.8047	0.4389	0.3269	0.066*	
C12	0.6454 (2)	0.43775 (9)	0.1093 (2)	0.0676 (5)	
H12	0.6392	0.4860	0.1000	0.081*	
C13	0.5681 (2)	0.33184 (9)	0.0080 (2)	0.0604 (4)	
H13	0.5069	0.3043	−0.0741	0.073*	
C14	0.66414 (18)	0.29849 (8)	0.14078 (18)	0.0476 (3)	
H14	0.6667	0.2502	0.1475	0.057*	
H2'	0.944 (2)	0.2338 (8)	0.2948 (14)	0.053 (4)*	

### Atomic displacement parameters (Å^2^)

**Table:**

	*U*^11^	*U*^22^	*U*^33^	*U*^12^	*U*^13^	*U*^23^
F1	0.0844 (14)	0.0480 (10)	0.0645 (11)	−0.0090 (8)	−0.0157 (9)	−0.0032 (8)
F1B	0.129 (3)	0.0477 (14)	0.0729 (16)	−0.0225 (13)	−0.0185 (15)	0.0026 (11)
O1	0.0791 (8)	0.0563 (7)	0.0350 (5)	0.0115 (5)	−0.0015 (5)	−0.0073 (4)
N1	0.0442 (6)	0.0490 (6)	0.0280 (5)	0.0044 (5)	0.0026 (4)	0.0053 (5)
N2	0.0477 (7)	0.0498 (7)	0.0248 (5)	0.0072 (5)	0.0021 (4)	0.0029 (4)
N3	0.0639 (9)	0.0691 (9)	0.0618 (8)	0.0087 (7)	−0.0132 (7)	0.0126 (7)
C1	0.0514 (8)	0.0522 (8)	0.0421 (7)	0.0001 (6)	−0.0015 (6)	0.0027 (6)
C2	0.0646 (11)	0.0874 (13)	0.0435 (8)	−0.0005 (9)	−0.0116 (7)	0.0025 (8)
C3	0.0748 (12)	0.0848 (13)	0.0540 (10)	0.0174 (10)	−0.0023 (9)	0.0261 (9)
C4	0.0912 (14)	0.0584 (10)	0.0680 (11)	0.0085 (9)	0.0046 (10)	0.0226 (9)
C5	0.0708 (11)	0.0504 (9)	0.0540 (9)	−0.0012 (8)	0.0025 (8)	0.0085 (7)
C6	0.0409 (7)	0.0462 (7)	0.0356 (6)	0.0027 (6)	0.0043 (5)	0.0051 (5)
C7	0.0413 (7)	0.0444 (7)	0.0328 (6)	−0.0021 (5)	0.0021 (5)	0.0031 (5)
C8	0.0755 (11)	0.0520 (8)	0.0381 (7)	0.0112 (8)	−0.0038 (7)	−0.0036 (6)
C9	0.0458 (7)	0.0446 (7)	0.0307 (6)	−0.0009 (5)	0.0017 (5)	0.0009 (5)
C10	0.0393 (7)	0.0440 (7)	0.0334 (6)	0.0040 (5)	0.0050 (5)	0.0009 (5)
C11	0.0611 (10)	0.0434 (8)	0.0539 (9)	−0.0009 (6)	−0.0071 (7)	0.0002 (6)
C12	0.0733 (12)	0.0476 (9)	0.0741 (11)	0.0041 (8)	−0.0117 (9)	0.0125 (8)
C13	0.0566 (9)	0.0658 (10)	0.0514 (9)	0.0039 (8)	−0.0139 (7)	−0.0045 (8)
C14	0.0492 (8)	0.0455 (7)	0.0452 (7)	0.0031 (6)	−0.0013 (6)	−0.0030 (6)

### Geometric parameters (Å, º)

**Table:**

F1—C1	1.288 (2)	C3—C4	1.358 (3)
F1B—C5	1.258 (2)	C4—C5	1.3782 (19)
O1—C9	1.2208 (16)	C5—C6	1.3856
N1—C7	1.2748 (13)	C6—C7	1.4846
N1—N2	1.3818 (15)	C7—C8	1.4977
N2—C9	1.3483 (18)	C9—C10	1.4951 (18)
N3—C13	1.321 (2)	C10—C11	1.376 (2)
N3—C12	1.325 (2)	C10—C14	1.380 (2)
C1—C6	1.3777 (16)	C11—C12	1.378 (2)
C1—C2	1.378 (2)	C13—C14	1.383 (2)
C2—C3	1.374 (3)		
			
C7—N1—N2	118.60 (9)	C5—C6—C7	121.8
C9—N2—N1	117.10 (10)	N1—C7—C6	115.31 (5)
C13—N3—C12	116.19 (14)	N1—C7—C8	126.29 (5)
F1—C1—C6	119.78 (13)	C6—C7—C8	118.4
F1—C1—C2	117.28 (16)	O1—C9—N2	123.57 (12)
C6—C1—C2	122.72 (15)	O1—C9—C10	120.04 (12)
C3—C2—C1	119.32 (17)	N2—C9—C10	116.35 (11)
C4—C3—C2	119.98 (16)	C11—C10—C14	118.00 (13)
C3—C4—C5	119.64 (16)	C11—C10—C9	119.08 (12)
F1B—C5—C4	116.26 (16)	C14—C10—C9	122.78 (12)
F1B—C5—C6	121.17 (12)	C10—C11—C12	118.87 (15)
C4—C5—C6	122.56 (10)	N3—C12—C11	124.12 (16)
C1—C6—C5	115.77 (7)	N3—C13—C14	124.51 (15)
C1—C6—C7	122.44 (7)	C10—C14—C13	118.29 (14)
			
C7—N1—N2—C9	−179.39 (11)	C5—C6—C7—N1	136.27 (7)
F1—C1—C2—C3	−174.5 (2)	C1—C6—C7—C8	136.79 (9)
C6—C1—C2—C3	0.1 (3)	C5—C6—C7—C8	−43.6
C1—C2—C3—C4	0.1 (3)	N1—N2—C9—O1	1.4 (2)
C2—C3—C4—C5	0.1 (3)	N1—N2—C9—C10	−176.38 (11)
C3—C4—C5—F1B	−179.8 (2)	O1—C9—C10—C11	37.3 (2)
C3—C4—C5—C6	−0.6 (2)	N2—C9—C10—C11	−144.86 (14)
F1—C1—C6—C5	173.92 (14)	O1—C9—C10—C14	−138.39 (15)
C2—C1—C6—C5	−0.56 (18)	N2—C9—C10—C14	39.45 (18)
F1—C1—C6—C7	−6.5 (2)	C14—C10—C11—C12	−0.5 (2)
C2—C1—C6—C7	179.04 (12)	C9—C10—C11—C12	−176.42 (15)
F1B—C5—C6—C1	180.0 (2)	C13—N3—C12—C11	0.6 (3)
C4—C5—C6—C1	0.80 (13)	C10—C11—C12—N3	−0.1 (3)
F1B—C5—C6—C7	0.39 (19)	C12—N3—C13—C14	−0.4 (3)
C4—C5—C6—C7	−178.81 (11)	C11—C10—C14—C13	0.7 (2)
N2—N1—C7—C6	−177.77 (8)	C9—C10—C14—C13	176.42 (13)
N2—N1—C7—C8	2.13 (14)	N3—C13—C14—C10	−0.2 (3)
C1—C6—C7—N1	−43.30 (10)		

### Hydrogen-bond geometry (Å, º)

**Table:**

*D*—H···*A*	*D*—H	H···*A*	*D*···*A*	*D*—H···*A*
N2—H2′···N1^i^	0.87 (1)	2.45 (1)	3.1420 (15)	137 (1)
N2—H2′···O1^i^	0.87 (1)	2.38 (1)	3.1777 (15)	154 (2)
C8—H8*C*···F1^i^	0.96	2.46	3.1603 (19)	129
C8—H8*C*···O1^i^	0.96	2.58	3.0680 (13)	112
C13—H13···F1^ii^	0.93	2.34	3.238 (2)	161
C14—H14···O1^i^	0.93	2.50	3.1849 (19)	131

Symmetry codes: (i) *x*, −*y*+1/2, *z*−1/2; (ii) *x*−1, *y*, *z*−1.
